# The effects of a pharmacist-led medication review in a nursing home: A randomized controlled trial

**DOI:** 10.1097/MD.0000000000028023

**Published:** 2021-12-03

**Authors:** Wen-Shyong Liou, Shih-Ming Huang, Wei-Hsin Lee, Yen-Lin Chang, Ming-Fen Wu

**Affiliations:** a Department of Pharmacy, Taichung Veterans General Hospital, Taichung, Taiwan, R.O.C.; b School of Pharmacy, China Medical University, Taichung, Taiwan, R.O.C.

**Keywords:** elderly, medication reconciliation, nursing home, pharmaceutical care

## Abstract

**Background:**

In this study, an intensive review of pharmaceutical care for elderly patients was conducted in a Veterans Administration nursing home in Taiwan and its effects were evaluated.

**Methods:**

One hundred participants were enrolled in this randomized controlled study with even distribution. The inclusion criteria were age 65 years or older, prescriptions for at least 5 oral medicines daily, and ≥2 chronic diseases, for the period May 2013 to October 2014. Subjects were excluded if they had previously been included in an intensive medication review conducted by a pharmacist. The primary outcomes were numbers of drugs prescribed, potential inappropriate medications, and numbers of drug-related problems. The secondary outcomes were self-reported medical usages, measurements of quality of life, results of a satisfaction survey, and health status.

**Results:**

A total of 80 cases (42 in the intervention group with medication reconciliation and 38 in the control group without medication reconciliation) completed the study. Baseline characteristics were not statistically different between the 2 groups. The overall prevalence of potential inappropriate medication was 74.3%. There were no differences between the 2 groups, with the exception of “medical problems,” which showed a significantly higher prevalence in the intervention group (*P* < .05). The intervention group reported greater satisfaction regarding pharmacist visits and medication compliance (*P* < .01). The mean number of drug-related problems was significantly lower after the intervention (*P* < .01).

**Conclusion:**

In this study, the intensive review of the elderly patients’ medications revealed that the only significant effect of pharmaceutical care was on “all outcomes.” A possible reason for this is the rather advanced ages of some patients who needed a considerable number of medications to treat several chronic diseases. Another reason may be the small sample size. However, participants who received the pharmacist intervention did have higher satisfaction with medication reconciliation and fewer drug-related problems.

## 1 Introduction

Taiwan's population is aging rapidly and this follows a global trend.^[1–2]^ Elderly patients frequently suffer from one or more chronic diseases as a result of pathophysiological changes, and thus they may receive treatment with a variety of healthcare technologies which often involve exposure to polypharmacy.^[3–6]^ Inappropriate prescriptions due to polypharmacy can increase the risk of adverse drug events, hospitalization, multi-morbidities, and mortality. Drugs with an unfavorable risk/benefit ratio, uncertain therapeutic effects, or ones that can be replaced by safer alternatives are termed potentially inappropriate medications (PIMs). The potential prevalence of PIMs ranged from 8.5% to 50%.^[2,7–10]^ Studies have shown that reducing PIMs improved health outcomes and quality of life, decreased hospital administration, and lowered medical expenditure.^[10–12]^

The Beers criteria, first published in 1991, were developed to discourage the use of PIMs in older adults.^[13]^ This well-defined list, which has been updated and revised several times, has been widely adopted in many countries as an indicator for quality of geriatric healthcare.^[14,15]^ Medication reviews using the Beers criteria to show the effect of medication reconciliation or pharmaceutical care are conducted by pharmacists or other healthcare professionals, and are widely applied in clinical settings.

Pharmacist participation has been shown to improve the quality of medication reconciliation in a number of studies.^[10,16–21]^ Pharmacists can identify more nonprescription and herbal medications than physicians or nurses using the medication reconciliation approach when patients are admitted to hospital.^[16]^ Medication reconciliation conducted by clinical pharmacists may also significantly improve the health-related quality of life (HRQoL) among elderly patients.^[17]^ Most studies on medication reconciliation by pharmacists were shown to be effective or beneficial. However, the overall results of medication reviews or medication reconciliation remain controversial due to different study designs, research settings, consensus of the pharmaceutical intervention, institutional settings, and the ages of the studied populations.^[10,18–21]^ The majority of such studies are undertaken in a hospital setting. To date, few studies have been conducted on community-dwelling elders, such as patients in nursing homes, using a randomized controlled design, especially in East Asia.

In this pilot randomized controlled study, the use of clinical medication reviews via pharmacist visits was investigated to determine whether it could improve the HRQoL and clinical outcomes of residents in a Veterans Administration (VA) nursing home. By implementing home visits, pharmacists were better able to develop and apply strategies for managing subjects’ medications.

## 2 Methods

### 2.1 Study design

This randomized controlled study recruited patients aged 65 years old and over living in a long-term care facility in central Taiwan (Changhua Domiciliary Center where the location is closed to study hospital) run by the Veterans Affairs Commission, Executive Yuan, Taiwan, Republic of China. All residents who were prescribed at least 5 oral medicines daily and had 2 or more chronic diseases from May 2013 to October 2014 were voluntary enrolled. Subjects were excluded from the study if they had previously been included in a project involving intensive medication review by a pharmacist. Eligible residents were briefed about the study objectives and invited to participate in this 18-month study. After baseline data had been collected, patients were simply randomized using the computerized random number function in Microsoft Excel to receive either intensive medication review (intervention group) or usual care (control group, without a medication review by a visiting pharmacist). This study was approved by the Institutional Review Board of Taichung Veterans General Hospital, Taichung, Taiwan (CE13096) and the protocol was registered at a clinical trial website (NCT01823757). An informed consent form (ICF) was signed by each participant.

### 2.2 Clinical information

Subjects living in the facility were cared for by trained nurses. Basic information was recorded at regular intervals by the nurses, including a comprehensive geriatric assessment. The following information was collected from both groups: activities of daily living (ADL), instrumental ADL (IADL), mini-mental state examination (MMSE), health-related quality of life (HRQoL, including EuroQol 5 dimensions 3 levels [EQ-5D-3L] and EuroQol visual analogous scale [EQ-VAS]), as well as medical problems assessed by case managers over the research timeframe.

### 2.3 The procedure of pharmaceutical care

In addition to collection of baseline clinical information, eligible subjects in the intervention group were offered pharmaceutical services at months 1, 3, 7, and 13 (visit 1–visit 4), and related information was collected at month 18 (visit 5), while the control group was only visited at months 0 and 18 to obtain PIMs and overall numbers of drugs prescribed during the entire study period. The 13 pharmacists involved in this study were trained to employ a unification intervention approach, which included completing the medication administration record, assessing medication appropriateness of prescriptions, surveying the utilization of healthcare resources, and identifying drug-related problems (DRPs) in the intervention group.

Once medication administration problems were found, residents were educated in order to provide accurate knowledge related to administration of medications, and were provided with 7-day dosing administration aids, a pill splitter, as well as a personal health journal. Unless there were immediate life-threatening concerns, medication recommendations for DRPs were conveyed to physicians by medical chart notes. Patients, nurses, and nurse assistants were instructed to remove expired drugs and to report any adverse drug reactions or interactions to physicians. Due to the nature of the study design and intervention approach, participants in the control group were informed which group they were in after randomization, and continued to receive usual care from their original healthcare professionals.

The residents in the intervention group were requested to complete a 5-item questionnaire scored on a 10-point Likert scale to rate their level of medication knowledge, adherence, improvement of diseases, concerns about polypharmacy, and satisfaction with the pharmaceutical services at each visit. The control group only completed the questionnaire at the first and last visits.

### 2.4 Statistical analysis

The primary outcomes were the numbers of drugs prescribed, PIMs, and DRPs.^[22]^ The secondary outcomes included ADL/IADL, MMSE, EQ-5D-3L, and EQ-VAS based on the 2012 Beers Criteria, satisfaction survey, self-reported total numbers of outpatient visits, hospitalization, and emergency admissions.

As this was a pilot study, it was not appropriate to power the study. The appropriate powered sample size was calculated based on the initial population's mean difference in the baseline of EQ-5D-3L usual activity score between each group. Statistical analysis was done using 2-tailed tests and an α error of 0.05. For continuous variables, the Mann–Whitney *U* test was used. The power was 86.4% with 50 subjects in each group using the G∗Power program

The data are expressed as the mean ± SD for continuous variables, or as counts and proportions for categorical variables. Two groups were compared using the Mann–Whitney *U* test and Wilcoxon signed-ranks test. For data that were normally distributed (numbers of drugs prescribed), the independent sample *t* test was used. The Friedman test, a nonparametric version of the repeated measures ANOVA, was performed to investigate any decrease in DRPs due to the pharmacist intervention. Statistical significance was defined as a *P* value of .05. All of the analyses were performed using SPSS 22.0 (IBM, New York, NY).

## 3 Results

**Figure 1:**
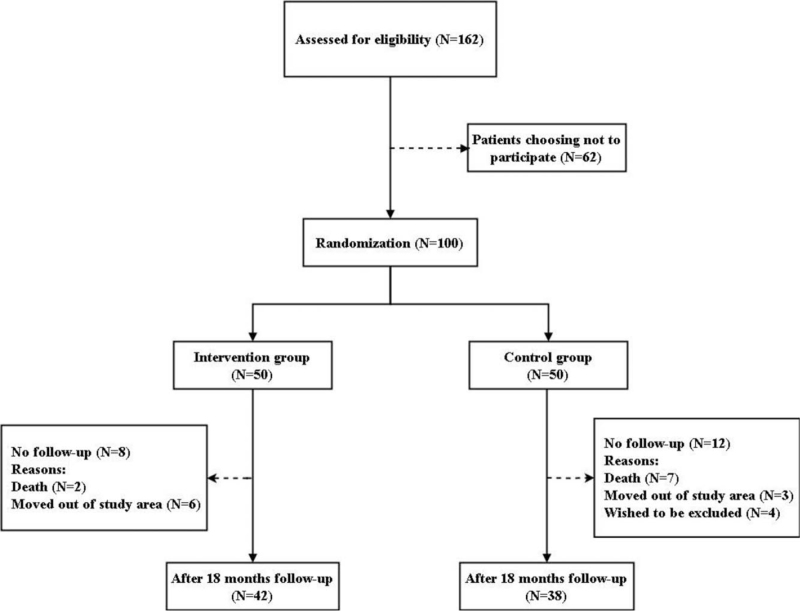
Study flow chart of the inclusion and assessment process.

One hundred residents met the inclusion criteria and were randomly assigned to the intervention group (n = 50) or the control group (n = 50). A total of 80 cases (42 in the intervention group and 38 in the control group) completed the 18-month study (the flow chart is shown in Fig. 1). There were no significant differences in the participants’ baseline demographic and clinical characteristics between the 2 groups (Table 1). The overall prevalence of PIMs was 74.3% (Table 2).

**Table 1:** Baseline demographic characteristics and medicines used.

	Intervention group (n = 50)	Control group (n = 50)	
Variables	Mean SD	Mean SD	*P* value
Age	86.7 ± 5.6	85.7 ± 3.6	.099
ADL	93.5 ± 8.4	93.8 ± 7.7	.984
IADL	6.8 ± 1.7	7.2 ± 1.1	.492
MMSE	24.9 ± 4.9	25.2 ± 3.9	.983
EQ-5D-3L			
Mobility	1.1 ± 0.4	1.1 ± 0.3	.357
Self-care	1.0 ± 0.2	1.0 ± 0.0	.160
Usual activity	1.2 ± 0.4	1.0 ± 0.2	.085
Pain/discomfort	1.2 ± 0.4	1.3 ± 0.5	.557
Anxiety/depression	1.1 ± 0.2	1.0 ± 0.2	.665
EQ-VAS	62.1 ± 9.0	60.9 ± 7.8	.959
Medical problems	4.4 ± 1.7	4.7 ± 2.0	.538
Outpatient visits	3.8 ± 2.9	4.4 ± 3.3	.410
Hospitalization admissions	0.1 ± 0.3	0.2 ± 0.6	.658
Emergency admissions	0.2 ± 0.4	0.2 ± 0.5	.853
Numbers of drug prescribed^a^	9.9 ± 3.3	10.3 ± 2.9	.610
Self-reported medication usage			
OTC drugs used	0.6 ± 0.8	0.6 ± 1.4	.574
Herbal medicines used	0.1 ± 0.6	0.1 ± 0.4	.739
Numbers of potentially inappropriate medications	1.2 ± 1.0	1.0 ± 0.8	.260

**Table 2:** Numbers of potentially inappropriate medications (PIMs) at each visit.

	Total participants	Participants who had a PIM	PIM%
Visit 0-intervention	50	36	72.0%
Visit 0-control	50	37	74.0%
Visit 1	49	36	73.5%
Visit 2	43	32	74.4%
Visit 3	43	33	76.7%
Visit 4	42	31	73.8%
Visit 5-intervention	42	32	76.2%
Visit 5-control	38	28	73.7%

### 3.1 Changes in clinical outcomes

The number of medical problems that the participants were aware of was significantly higher in the intervention group (3.0 ± 4.0 vs 0.9 ± 2.7, *P* < .05) at the end of the study, whereas there were no significant differences in the numbers of drugs prescribed, PIMs, healthcare utilization, and quality of life between the groups (Table 3).

**Table 3:** The mean changes of outcome variables within 18 months.

	Intervention group (n = 42)	Control group (n = 38)	
	Mean SD	Mean SD	*P* value
ADL	−5.0 ± 8.3	3.8 ± 29.4	.186
IADL	−1.3 ± 1.5	−1.1 ± 1.9	.894
MMSE	−2.0 ± 6.3	−2.3 ± 8.2	.928
EQ-5D-3L			
Mobility	0.3 ± 0.5	0.5 ± 0.6	.305
Self-care	0.1 ± 0.4	0.2 ± 0.4	.670
Usual activity	0.1 ± 0.5	0.1 ± 0.4	.749
Pain/discomfort	0.0 ± 0.6	−0.1 ± 0.5	.676
Anxiety/depression	0.0 ± 0.4	0.1 ± 0.4	.513
EQ-VAS	−7.5 ± 24.6	1.2 ± 24.4	.182
Medical problems	3.0 ± 4.0	0.9 ± 2.7	.035^∗^
Outpatient visits	0.5 ± 3.1	−0.2 ± 4.0	.342
Hospitalization admissions	0.0 ± 0.2	−0.1 ± 0.8	.931
Emergency admissions	0.0 ± 0.5	−0.1 ± 0.7	.334
Numbers of drug prescribed^a^	−1.0 ± 3.6	−0.6 ± 3.8	.700
Self-reported medication usage			
OTC drugs used	−0.4 ± 0.8	−0.6 ± 1.6	.983
Herbal medicines used	−0.1 ± 0.3	−0.1 ± 0.4	.578
Numbers of PIMs	−0.2 ± 1.0	0.0 ± 1.0	.481

### 3.2 Drug-related problems

**Figure 2:**
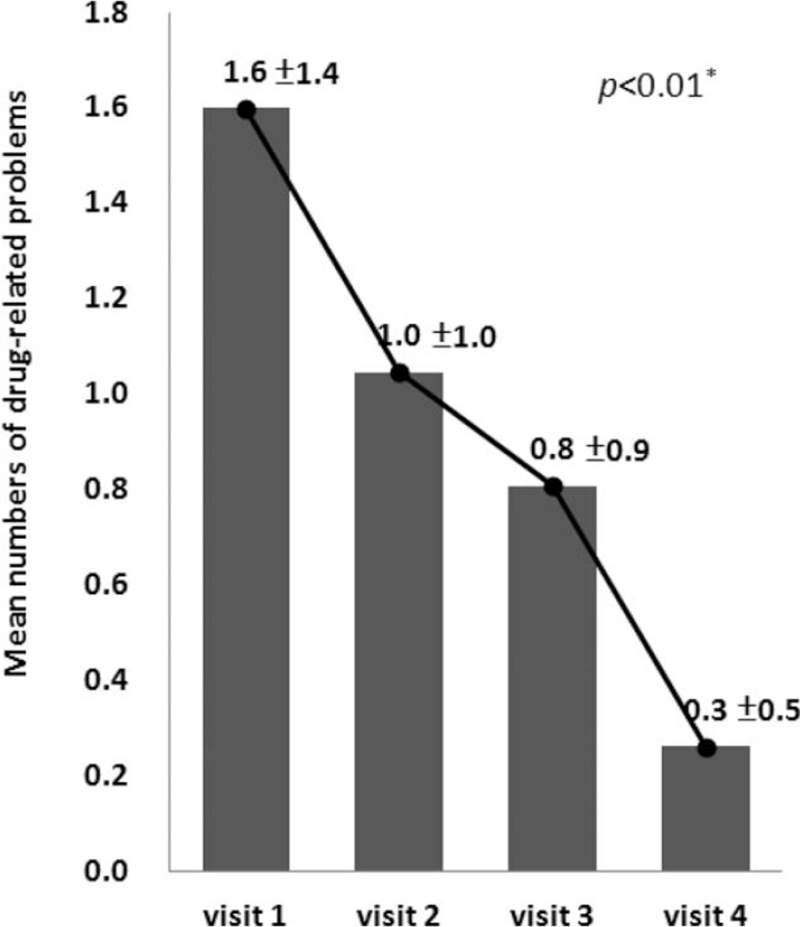
Mean numbers of drug-related problems (DRPs) at each visit in the intervention group.

There were 158 DRPs that occurred during the entire study period in the intervention group participants who completed the study. The mean number of DRPs for the intervention group dropped significantly from 1.6 ± 1.4 (visit 1) to 0.3 ± 0.5 at the end of the study (*P* < .01, Fig. 2). The 3 most common patient-associated DRPs (Table 4) were incorrect concept of taking medications (27.9%, including withholding medications, doubling doses arbitrarily, etc), need to perform therapeutic drug monitoring (TDM, 13.9%), and inappropriate storage (9.8%). The 3 most common physician-associated DRPs were duplicated medication (19.4%), existence of more suitable medications (13.9%), and dose too high (11.1%). The overall response rate for physicians and patients who responded to the pharmacists’ recommendations was 96.8%, with an acceptance rate of 77.2% (Table 5).

**Table 4:** Classification of drug-related problems (total DRPs number = 158).

	Physician-associated DRPs		Patient-associated DRPs		Total DRPs	
Code (detailed classification)	n	%	n	%	n	%
1 Addition of a medication	**5**	**(13.9%)**	**2**	**(1.6%)**	**7**	**(4.4%)**
11 Untreated acute situation or diseases	2	(5.6%)	1	(0.8%)	3	(1.9%)
12 Need preventable medication	1	(2.8%)	0	(0.0%)	1	(0.6%)
13 Need to add other medications to enhance efficacy	0	(0.0%)	1	(0.8%)	1	(0.6%)
14 Need medication for chronic diseases	2	(5.6%)	0	(0.0%)	2	(1.3%)
2 Delete current medications	**8**	**(22.2%)**	**9**	**(7.4%)**	**17**	**(10.8%)**
21 Off-label use	0	(0.0%)	2	(1.6%)	2	(1.3%)
22 Duplicated medication	7	(19.4%)	6	(4.9%)	13	(8.2%)
23 Needs no medication	0	(0.0%)	1	(0.8%)	1	(0.6%)
24 To treat avoidable adverse drug reaction from other medications	1	(2.8%)	0	(0.0%)	1	(0.6%)
3 Inappropriate medications	**8**	**(22.2%)**	**1**	**(0.8%)**	**9**	**(5.7%)**
31 Inappropriate dosage form	2	(5.6%)	0	(0.0%)	2	(1.3%)
33 Incompatibility	1	(2.8%)	0	(0.0%)	1	(0.6%)
35 More suitable medications exist	5	(13.9%)	0	(0.0%)	5	(3.2%)
38 Medication selected previously failed	0	(0.0%)	1	(0.8%)	1	(0.6%)
4 Low dosing	**2**	**(5.6%)**	**16**	**(13.1%)**	**18**	**(11.4%)**
41 Dose too low or low blood concentration	1	(2.8%)	3	(2.5%)	4	(2.5%)
45 Wrong administration route	0	(0.0%)	1	(0.8%)	1	(0.6%)
46 Inappropriate storage	1	(2.8%)	12	(9.8%)	13	(8.2%)
5 High dosing	**5**	**(13.9%)**	**2**	**(1.6%)**	**7**	**(4.4%)**
51 Dose too high	4	(11.1%)	1	(0.8%)	5	(3.2%)
55 Poor renal or hepatic function	1	(2.8%)	1	(0.8%)	2	(1.3%)
6 Adverse drug reaction	**6**	**(16.7%)**	**13**	**(10.7%)**	**19**	**(12.0%)**
61 Drug–drug interaction	1	(2.8%)	3	(2.5%)	4	(2.5%)
64 Not safe for the patients (due to some risk factors)	1	(2.8%)	0	(0.0%)	1	(0.6%)
65 Unexpected pharmacological reaction under normal dose	3	(8.3%)	6	(4.9%)	9	(5.7%)
66 Unsafe medications	1	(2.8%)	0	(0.0%)	1	(0.6%)
67 Idiosyncrasy	0	(0.0%)	2	(1.6%)	2	(1.3%)
68 Incorrect administration	0	(0.0%)	2	(1.6%)	2	(1.3%)
9 Poor medication adherence	**2**	**(5.6%)**	**69**	**(56.6%)**	**71**	**(44.9%)**
91 Complicated dosing schedules	0	(0.0%)	2	(1.6%)	2	(1.3%)
92 Incorrect concept of taking medications	1	(2.8%)	34	(27.9%)	35	(22.2%)
94 Unaware of correct way to take medications	0	(0.0%)	11	(9.0%)	11	(7.0%)
95 Frequently forgot to take medication	0	(0.0%)	4	(3.3%)	4	(2.5%)
96 Unable to swallow	1	(2.8%)	1	(0.8%)	2	(1.3%)
97 Needs therapeutic drug monitoring	0	(0.0%)	17	(13.9%)	17	(10.8%)
Other	**0**	**(0.0%)**	**10**	**(8.2%)**	**10**	**(6.3%)**
	0	(0.0%)	10	(8.2%)	10	(6.3%)
Total	**36**	**(100.0%)**	**122**	**(100.0%)**	**158**	**(100.0%)**

**Table 5:** Response and acceptance rate of DRPs after pharmacists’ intervention (n = 158).

	Physician-associated DRPs	Patient-associated DRPs
Number of drugs needed in pharmacist intervention	36	122
Response rate	100%	95.9%
Acceptance rate	69.4%	79.5%

### 3.3 Satisfaction survey

Participants in the intervention group had significantly higher scores than those in the control group with respect to following their doctor's instruction to take medicines (10.0 ± 0.0 vs 8.7 ± 2.1, *P* < .05) and willingness to receive further visits by a pharmacist (8.9 ± 2.2 vs 7.4 ± 3.1, *P* < .005, Table 6). Within-groups analyses indicated similar results (Table 7).

**Table 6:** The outcomes of satisfaction survey after pharmacist intervention.

	First visit			After 18 months		
	Intervention (n = 50)	Control (n = 50)		Intervention (n = 42)	Control (n = 38)	
	Mean SD	Mean SD	*P* value	mean SD	mean SD	*p* value
Do you follow the doctor's instruction to take medications each day?	8.2 ± 2.5	8.7 ± 1.9	.484	10.0 ± 0.0	8.7 ± 2.1	.000^∗^
Do you feel better after taking your medications?	7.4 ± 2.1	6.9 ± 2.0	.170	7.1 ± 2.4	6.8 ± 2.4	.594
Do you worry about receiving too many medications?	4.6 ± 3.1	4.8 ± 3.1	.731	4.4 ± 3.7	5.7 ± 3.8	.079
Are you aware of all of your medications?	7.0 ± 3.2	7.0 ± 3.4	.794	7.4 ± 3.6	7.4 ± 2.9	.354
Are you willing to receive further visits by a pharmacist?	7.3 ± 2.5	7.4 ± 2.4	.734	8.9 ± 2.2	7.4 ± 3.1	.004^∗^

**Table 7:** The outcomes of satisfaction survey, within-groups comparison (n = 80).

	Intervention (n = 42)			Control (n = 38)		
	First visit	After 18 months		First visit	After 18 months	
	Mean SD	Mean SD	*P* value	Mean SD	Mean SD	*P* value
Do you follow the doctor's instruction to take medications each day?	8.3 ± 2.4	10.0 ± 0.0	.000^∗^	8.6 ± 2.1	8.7 ± 2.1	.728
Do you feel better after taking your medications?	7.5 ± 2.2	7.1 ± 2.4	.453	6.7 ± 2.1	6.8 ± 2.4	.804
Do you worry about receiving too many medications?	4.6 ± 3.3	4.4 ± 3.7	.986	4.9 ± 3.0	5.7 ± 3.8	.458
Are you aware of all of your medications?	7.4 ± 2.8	7.4 ± 3.6	.983	6.8 ± 3.5	7.4 ± 2.9	.402
Are you willing to receive further visits by a pharmacist?	7.2 ± 2.6	8.9 ± 2.2	.001^∗^	7.3 ± 2.7	7.4 ± 3.1	.466

## 4 Discussion

The aim of this study was to assess the effects of pharmacist-led medication reviews on the residents of a VA nursing home. In the intervention group, there was no significant difference in terms of the numbers of drugs prescribed, PIMs, health resource utilization, and HRQoL after pharmacist visits, compared with the control group. With an average age of over 85 years old, the residents enrolled in our study were primarily categorized into the oldest-old age group and thus tended to suffer from multiple comorbidities. Consequently, it was necessary to have realistic expectations as to the likelihood that the pharmacist intervention alone would be capable of having a marked effect on the patients’ medications and related outcomes. However, the intervention group demonstrated significant improvement in DRPs, self-reported adherence, and the degree of satisfaction with pharmaceutical care.

### 4.1 Health-related quality of life and basic functions

Daily activities, mental status, and HRQoLs were evaluated using several tools such as ADL, IADL, MMSE, EQ-5D-3L, and EQ-VAS, which worsened with age and showed no significant differences between the control and intervention groups. At each visit, the pharmacist only provided a medication review and consultation. Therefore, there was no direct and obvious improvement after the intervention. The findings of our study are consistent with a previous review which concluded that a pharmaceutical intervention had no apparent effect on hospitalizations, mortality, functional capacity, or cognitive functions.^[23]^

The number of medical problems was significantly higher in the intervention group at the end of the study. This can be explained, at least in part, by the declining nature of disease status in elderly patients over time. It is also reasonable to assume that participants in the intervention group understood their disease conditions better after repeated pharmacist visits, so they were likely more aware of concerning symptoms and, in turn, more likely to seek further medical care. Our findings are in line with other studies that showed medication reviews do not decrease health care utilization.^[24–26]^ However, the number of DRPs dropped significantly in our study, which was probably due to the pharmacists’ intervention.

### 4.2 Numbers of drug prescribed and potential inappropriate medications

The overall recommendation acceptance rate was 77.2%, which was lower compared with related studies.^[21,27,28]^ The fact that more recommendations were made for polypharmacy with a lower acceptance rate suggests that for patients receiving more medications an intervention may be less effective at improving the quality of prescribing.^[29]^ The complex regimens used in this study for the large proportion of oldest-old patients may account for the lower rate of physician acceptance.

Reducing the numbers of drugs prescribed and inappropriate prescribing are usually the main effects of pharmaceutical intervention, but the clinical significance of such improvements is unclear.^[23,30]^ The numbers of drugs prescribed and PIMs declined without a significant difference between the 2 groups at the end of the study. Studies on medication reconciliation or pharmacist intervention have yielded conflicting findings.^[10,18,19,31]^ A trend of decreasing PIMs in the intervention group was observed comparing the first and the last visit, which indicates a possible positive effect of the pharmaceutical intervention.

Alpha-blockers and benzodiazepines were the most common drug classes involved in PIMs in this study. One study evaluated medications prescribed for the frail elderly using the Beers criteria, and found that the most commonly prescribed PIMs were benzodiazepines (10%), whereas doxazosin accounted for a mere 1.4%.^[32]^ The difference is probably related to Taiwan's National Health Insurance policy, whereby a patient taking doxazosin for benign prostatic hyperplasia would be identified as receiving a duplicate medication if he also had a prescription for an antihypertensive agent. To avoid rejection of medication reimbursements, most health institutes in Taiwan prohibit the use of doxazosin for patients with hypertension.

### 4.3 Satisfaction survey

Significantly higher scores for following the doctor's instruction to take medicine, and willingness to receive visits by a pharmacist were found in the intervention group. This indicates an improvement in compliance as well as trust and satisfaction with the pharmacist, which would likely lead to a reduction in adverse drug reactions.^[33]^

### 4.4 Improvement of drug-related problems

A reduction in DRPs was the most obvious effect in this study. A lack of knowledge about the application of medications was identified during interviews with patients. Several residents misunderstood the dosing frequency or forgot to take medicines, but did not inform their doctors and adjusted doses themselves. The most common problem with respect to drug storage was related to eye drops. Residents would discard eye drops only when the bottles were exhausted. Subjects were directed to throw away the preparations 1 month after opening the bottles to prevent infections. Although the improvement in DRPs was not correlated with the numbers of drugs prescribed and hospital admissions, it certainly improved the accuracy of medications and safety. A “duplicated medication” was the most common physician-associated DRP. Several inappropriate prescriptions were modified according to recommendations, and the rest were evaluated by a pharmacist for possible adverse impacts.

### 4.5 Limitations

In this study, in our analysis of the primary outcomes we were only able to identify the difference in numbers of DRPs between the intervention group and the control group. Possible reasons for this include the small sample size, cultural factors, and characteristics of the nursing home. Without a sufficiently large sample size, analyses may lack the statistical power needed to identify significant differences in outcomes. This pilot study had limited funding and thus it was not possible to conduct a large-scale study. The participants were all male veterans and this may explain, at least in part, why the results were non-significant, whereas a nursing home with both women and men residents may yield different results. The nursing home is run by the Veterans Administration of Taiwan. Physicians, nurses, and pharmacists in the facility provide residents with usual care. Medical staff may be more inclined to adhere to the familiarity of standard practice rather than implement recommendations, which would explain why the numbers of drugs prescribed and PIMs did not change after the clinical pharmacists intervened. Nevertheless, numbers of DRPs were significantly reduced, which means the pharmacists’ intervention did indeed confer beneficial effects on the elderly residents.

Caring for the elderly, especially the oldest-old, is a challenging issue in many aged societies. We believe that a multidisciplinary health team, one that includes a physician, a nurse, and a pharmacist, is essential to providing elderly patients with optimal pharmacotherapy. This pilot randomized controlled study is the first of its kind to be conducted in Taiwan's VA system and, to the best of our knowledge, is the first RCT on elderly patients in East Asia to evaluate the effects of pharmacist intervention. We hope these findings will help to inform future VA systematic and strategic planning for residential care in Taiwan's VA nursing homes.

## 5 Conclusion

In this 18-month study of pharmaceutical care in a nursing home, which employed patient interviews, medication reviews, and recommendations for DRPs, there were no obvious differences between the intervention and control groups in basic functions, HRQoL, utilization of health resources, and the numbers of drugs prescribed at the end of the study, but the number of PIMs decreased. Residents in the intervention group showed improvements in recognizing health status, caring for themselves, following their doctor's instructions, and satisfaction with the pharmacist, and numbers of DRPs were significantly reduced. With the recommendation acceptance rate of residents approaching 80%, the accuracy of applying medication was better in the intervention group. The average age of patients was over 85 years, and therefore they tended to have a variety of complex medical problems, which likely limited any improvements achieved by the pharmacists’ pharmaceutical intervention. This pilot study may serve as the basis for future research and may contribute to improvements in the clinical care of residents in Taiwan's VA nursing homes.

## Acknowledgments

The authors are grateful to the pharmacists, physicians, and nursing home staff, and residents who took part in this study. They also express their thanks to the Biostatistics Task Force of Taichung Veterans General Hospital for assisting with the statistical analysis.

## Author contributions

**Conceptualization:** Wen-Shyong Liou.

**Data curation:** Wei-Hsin Lee, Yen-Lin Chang, Ming-Fen Wu.

**Formal analysis:** Yen-Lin Chang.

**Funding acquisition:** Wen-Shyong Liou.

**Investigation:** Wen-Shyong Liou, Shih-Ming Huang, Wei-Hsin Lee.

**Methodology:** Wen-Shyong Liou.

**Project administration:** Ming-Fen Wu.

**Supervision:** Wen-Shyong Liou, Ming-Fen Wu.

**Validation:** Wen-Shyong Liou.

**Visualization:** Wen-Shyong Liou.

**Writing – original draft:** Wen-Shyong Liou, Shih-Ming Huang.

**Writing – review & editing:** Wen-Shyong Liou.
